# Bacteriophage endolysin Ply113 as a potent antibacterial agent against polymicrobial biofilms formed by *enterococci* and *Staphylococcus aureus*

**DOI:** 10.3389/fmicb.2023.1304932

**Published:** 2023-12-12

**Authors:** Jing Wang, Siyu Liang, Xiaofeng Lu, Qiu Xu, Yao Zhu, Shenye Yu, Wanjiang Zhang, Siguo Liu, Fang Xie

**Affiliations:** State Key Laboratory for Animal Disease Control and Prevention, Harbin Veterinary Research Institute, Chinese Academy of Agricultural Sciences, Harbin, China

**Keywords:** bacteriophage endolysin, *Enterococcus faecium*, *Enterococcus faecalis*, *Staphylococcus aureus*, antibacterial activity, antibiofilm activity, polymicrobial biofilms

## Abstract

Antibiotic resistance in *Enterococcus faecium*, *Enterococcus faecalis*, and *Staphylococcus aureus* remains a major public health concern worldwide. Furthermore, these microbes frequently co-exist in biofilm-associated infections, largely nullifying antibiotic-based therapy. Therefore, it is imperative to develop an efficient therapeutic strategy for combating infections caused by polymicrobial biofilms. In this study, we investigated the antibacterial and antibiofilm activity of the bacteriophage endolysin Ply113 *in vitro*. Ply113 exhibited high and rapid lytic activity against *E. faecium*, *E. faecalis*, and *S. aureus*, including vancomycin-resistant *Enterococcus* and methicillin-resistant *S. aureus* isolates. Transmission electron microscopy revealed that Ply113 treatment led to the detachment of bacterial cell walls and considerable cell lysis. Ply113 maintained stable lytic activity over a temperature range of 4–45°C, over a pH range of 5.0–8.0, and in the presence of 0–400 mM NaCl. Ply113 treatment effectively eliminated the mono-species biofilms formed by *E. faecium*, *E. faecalis*, and *S. aureus* in a dose-dependent manner. Ply113 was also able to eliminate the dual-species biofilms of *E. faecium*–*S. aureus* and *E. faecalis*–*S. aureus*. Additionally, Ply113 exerted potent antibacterial efficacy *in vivo*, distinctly decreasing the bacterial loads in a murine peritoneal septicemia model. Our findings suggest that the bacteriophage endolysin Ply113 is a promising antimicrobial agent for the treatment of polymicrobial infections.

## 1 Introduction

The Gram-positive bacteria *Enterococcus faecium*, *Enterococcus faecalis*, and *Staphylococcus aureus* are important opportunistic pathogens affecting both humans and animals, seriously threatening public health worldwide (Kadariya et al., 2014; García-Solache and Rice, 2019). These pathogens can cause severe and diverse clinical manifestations, such as bacteremia, endocarditis, urinary tract infections, skin wounds, and soft tissue infections (García-Solache and Rice, 2019; Cheung et al., 2021). Currently, an increasing number of antibiotic-resistant strains of *E. faecium*, *E. faecalis*, and *S. aureus* are being found in hospital and community-related infections, causing a huge economic burden globally (Jaradat et al., 2020; Van Hal et al., 2022). The emergence of vancomycin-resistant *Enterococcus* (VRE) and methicillin-resistant *S. aureus* (MRSA) poses significant challenges to clinical treatment. In recent years, MRSA infections have shifted from healthcare-related infections to community-related infections. These community-acquired strains carry the staphylococcal chromosome cassette *mec* IV, which is easily transferred horizontally, exacerbating the prevalence of MRSA (Ma et al., 2002; Long, 2020). Similarly, the prevalence of invasive VRE infections has recently increased worldwide, and they are difficult to treat, resulting in a high risk of mortality (Papanicolaou et al., 2019). Both VRE and MRSA have been highlighted by the World Health Organization, which has listed them on its most recent list of high-priority pathogens for which new therapies are urgently needed (Tacconelli et al., 2018).

*Enterococcus* species and *S. aureus* are known for their propensity to produce biofilms, which aids in their survival in hostile environments and persistence on host tissue (Moormeier and Bayles, 2017; Ch'ng et al., 2019). Biofilm-associated infections are not only difficult to eradicate, but they also serve as a breeding ground for bacteria. In comparison to infection with mono-species biofilms, infection with polymicrobial biofilms usually results in more severe clinical signs with a high mortality rate (Peters et al., 2012). *Enterococcus* species and *S. aureus*, especially VRE and MRSA, are frequently co-isolated from intestinal samples, chronic wounds, and device insertion sites of patients (Furuno et al., 2005; Heinze et al., 2019; Yoon et al., 2019). In the microenvironment of polymicrobial biofilms, VRE has been observed to promote the transfer of antibiotic resistance genes and the formation of vancomycin-resistant *S. aureus* (Weigel et al., 2007). Additionally, *S. aureus* could offer heme to boost *E. faecalis* growth and the formation of dual-species biofilms (Ch'ng et al., 2022). Therefore, new antibacterial agents need to be active not just against antibiotic-resistant bacteria, but also against infections caused by polymicrobial biofilms.

Bacteriophages and their derived lytic proteins are an intriguing class of therapeutic agents with significant potential in treating infections caused by antibiotic-resistant pathogens. Bacteriophages, also known as phages, are viruses that can infect and replicate in bacteria. When the lytic bacteriophages infect their host bacteria, they inject their genome into the host bacteria and use their host’s biosynthetic machinery to replicate and produce progeny virions (Rodriguez-Rubio et al., 2016). At the last phase of the lytic cycle, bacteriophage endolysins are generated to break the bacterial cell walls and allow the progeny to be released (Roach and Donovan, 2015). Whereas the lytic cycle results in bacterial cell lysis, the lysogenic cycle involves the phage genome integrating into the host bacterial genome and replicating alongside the host (Erez et al., 2017). Bacteriophage endolysins are widely regarded as a promising antibacterial alternative for treating antibiotic-resistant bacteria in clinical settings. Furthermore, several endolysins have been demonstrated to be effective in the control of biofilm formation, highlighting their potential as antibiofilm agents that could be used in the treatment of biofilm-associated infection (Sass and Bierbaum, 2007; Poonacha et al., 2017; Lusiak-Szelachowska et al., 2020).

In this study, we investigated the antibacterial activity of Ply113, an endolysin derived from an *E. faecium* bacteriophage, against antibiotic-resistant strains of *E. faecium*, *E. faecalis*, and *S. aureus*. The efficacy of Ply113 was also evaluated in eradicating mono-species and polymicrobial biofilms produced by *Enterococcus* species and *S. aureus*.

## 2 Materials and methods

### 2.1 Bacterial strains and growth conditions

The *E. faecium*, *E. faecalis*, and *S. aureus* strains used in this study are listed in Supplementary Table S1. All *E. faecium* and *E. faecalis* strains were routinely cultured in Brain Heart Infusion (BHI) broth (Difco, Detroit, MI, United States) at 37°C, and all *S. aureus* strains were grown in Tryptic Soy Broth (TSB) medium (Difco) at 37°C. *Escherichia coli* strains DH5α and BL21 (DE3) were cultured in Luria–Bertani (LB) broth (Difco) to clone and express the Ply113 protein, respectively.

### 2.2 Bioinformatics analysis

The conserved domains of Ply113 were identified using SMART (Letunic et al., 2021). Three-dimensional (3D) structural homology modeling of Ply113 was conducted with the automated SWISS-MODEL (Bienert et al., 2017). Molecular models were analyzed and presented by PyMOL (DeLano Scientific, Palo Alto, CA, United States).

### 2.3 Protein expression and purification

The gene fragment encoding Ply113 (GenBank Accession No. MZ147816.1) was synthesized by BGI Biotech (Beijing, China) and cloned into the pET22b expression vector (Novagen, San Diego, CA, USA). The recombinant plasmid containing the correct insert was transformed into *E. coli* BL21 (DE3) competent cells. To induce Ply113 protein expression, the cells were grown in LB broth to an optical density at 600 nm (OD_600_) of 0.6. Then, Isopropyl β-D-1-thiogalactopyranoside (IPTG) (Sigma-Aldrich, St. Louis, MO, United States) was added at a final concentration of 0.8 mM, and the cells were incubated at 16°C for 12 h. Bacterial cells were harvested by centrifugation at 6,000 × *g* for 10 min, washed twice, and then resuspended in 20 mL of lysis buffer (20 mM Tris–HCl, 500 mM NaCl, pH 8.0). After sonication on ice, the lysates were centrifuged at 13,000 × *g* for 10 min. The Ply113 protein was purified by loading the collected supernatant onto a Ni-Sepharose^™^ 6 Fast Flow column (GE Healthcare, Amersham, Uppsala, Sweden) according to the manufacturer’s instructions. Phosphate buffer saline (PBS) was used in place of elution buffer using a PD-10 desalination column (GE Healthcare). The purified Ply113 protein was verified using sodium dodecyl sulfate-polyacrylamide gel electrophoresis.

### 2.4 Determination of minimal inhibitory concentrations

The minimum inhibitory concentrations (MICs) of Ply113 and antibiotics were determined in triplicate by the standard broth microdilution method (Clinical and Laboratory Standards Institute [CLSI], 2012). Briefly, 5.0 × 10^5^ colony-forming units (CFU)/mL of freshly cultured *E. faecium*, *E. faecalis*, or *S. aureus* strains were distributed into the wells of 96-well polypropylene microtiter plates. Ply113, vancomycin, daptomycin, linezolid, and oxacillin were added to the wells in two-fold serial dilutions with final concentrations ranging from 0.5 to 256 μg/mL. The plate was incubated overnight at 37°C, and the MIC value was defined as the lowest concentration that inhibited visible growth.

### 2.5 Bactericidal assay

The bactericidal activity of Ply113 was determined by CFU reduction assays as previously described (Schuch et al., 2014). Briefly, the *E. faecium*, *E. faecalis*, and *S. aureus* strains were grown to an OD_600_ of 0.6 and collected by centrifugation at 3,000 × *g* for 10 min. The bacterial cells were washed three times with PBS, and then their concentration was adjusted to approximately 10^8^ CFU/mL. Ply113 was added to the bacterial suspension at a final concentration of 8 μg/mL. PBS buffer was used as a control. After treatment at 37°C for 1 h, the mixture was serially diluted and plated on BHI or TSB agar in triplicate. The CFU reduction was calculated following incubation at 37°C for 20 h. For the time-killing assay, *E. faecium* strain ATCC700221, *E. faecalis* strain ATCC51299, and *S. aureus* strain ATCC33591 were separately incubated with Ply113 and antibiotics (1 × MIC) at 37°C for 5 h. PBS buffer was used as a control. After incubation, each sample was diluted and spread onto BHI or TSB agar plates to count CFU. All assays were performed in triplicate.

### 2.6 Transmission electron microscopy

*E. faecium* strain ATCC700221, *E. faecalis* strain ATCC51299, and *S. aureus* strain ATCC33591 were grown in 10 mL of BHI or TSB medium at 37°C to the mid-logarithmic phase. The bacterial cells were washed with PBS and then treated with 4 μg/mL Ply113 for 1 h at 37°C. Bacteria cells treated with PBS were the control. The samples were fixed with 2.5% glutaraldehyde at 4°C overnight and then treated with 1% osmic acid at 4°C for 2 h, and the samples were dehydrated with a 50, 70, 90, and 100% (v/v) alcohol series. Then, the samples were embedded into epoxy resin overnight at 25°C, and polymerization was performed at 70°C for 48 h. Ultrathin sections (thicknesses ranging from 65 to 70 nm) were sliced and deposited on copper grids. After staining with uranyl acetate and lead citrate, morphology was observed under a transmission electron microscope (Hitachi, Tokyo, Japan).

### 2.7 Effects of temperature, salinity, and pH on Ply113 activity

To assess the effect of temperature on Ply113 lytic activity, a bacterial suspension of *E. faecalis* ATCC29212 (5.0 × 10^6^ CFU/mL) in PBS was treated with 4 μg/mL Ply113 and incubated at different temperatures ranging from 4°C to 45°C. The effect of salinity on Ply113 lytic activity was investigated by incubating the bacterial suspension in 20 mM Tris–HCl buffer (pH 7.0) with different NaCl concentrations (0–400 mM) at 37°C. For the pH stability assay, the bacterial suspension was treated with 4 μg/mL Ply113 in buffers with different pH ranges (50 mM sodium acetate for pH 5.0–6.0 and 20 mM Tris–HCl for pH 7.0–9.0). After 1 h of incubation, each sample was diluted and spread onto BHI or TSB agar plates to count CFU. The lytic activity was expressed as the relative reduction in bacterial CFU. The relative activity was determined by setting the maximum activity of Ply113 under various test conditions at 100%. The experiment was performed in triplicate.

### 2.8 Biofilm elimination assay in 24-well polystyrene plates

The effect of Ply113 against established biofilms of *E. faecium*, *E. faecalis*, and *S. aureus* was determined as previously described (Duarte et al., 2021). *E. faecium* D73, *E. faecalis* E141, and *S. aureus* YD2 were grown in TSB medium to the logarithmic growth phase. Then, 100 μL of bacterial culture was added to 900 μL of TSB medium in the wells of a 24-well polypropylene microtiter plate, which was incubated at 37°C for 24 h to induce the formation of mono-species biofilms. For the establishment of dual-species biofilms, *E. faecium* or *E. faecalis* culture was mixed with *S. aureus* culture at a 1:1 ratio, and 100 μL of the *E. faecium*–*S. aureus* mixture or *E. faecalis*–*S. aureus* mixture was added to 900 μL of TSB medium in 24-well plates to allow dual-species biofilm formation. For the biofilm elimination assay, the preformed biofilms were incubated with various concentrations of Ply113 (0–64 μg/mL) at 37°C for 2 h and washed with PBS. The remaining biofilms were stained with 0.1% crystal violet solution and dissolved in 33% acetic acid. The absorbance of the obtained solution in each well was measured at 570 nm using an EnVision Multimode Plate Reader (PerkinElmer, Waltham, United States). The number of culturable bacteria in the biofilm following Ply113 treatment was also quantified by counting CFU to evaluate the biofilm elimination efficiency of Ply113. All assays were performed in triplicate.

### 2.9 Confocal laser scanning microscopy

Cultures of *E. faecium* D73 or *E. faecalis* E141 in the logarithmic growth phase were mixed with *S. aureus* YD2 culture in a 1:1 ratio. Next, 200 μL of the *E. faecium*–*S. aureus* mixture or the *E. faecalis*–*S. aureus* mixture was added to 1.8 mL of TSB medium in a glass-bottom culture dish, which was incubated for 24 h at 37°C to induce the formation of dual-species biofilms. The established biofilms were washed twice with PBS and exposed to 8 μg/mL Ply113 for 2 h. Biofilms treated with PBS were the control. After Ply113 treatment, the biofilms were stained with the Live/Read^®^ BacLight^™^ kit (Invitrogen, Eugene, OR, United States) and observed using a confocal scanning laser microscope (Zeiss LSM 800, Jena, Germany).

### 2.10 Hemolysis assay

The *in vitro* hemolytic activity of Ply113 was determined as previously described (Tucker et al., 2018). First, 4% (w/v) red blood cells (RBCs) of pig and rabbit in PBS were incubated with various concentrations of Ply113 (ranging from 0 to 256 μg/mL) in 96-well polystyrene plates for 1 h at 37°C. RBC suspensions treated with 0.5% Triton X-100 and PBS were, respectively, used as positive and negative controls. The samples were centrifuged at 1,000 × *g* for 5 min, and the supernatants were transferred to new 96-well microplates. The absorbance was measured at 545 nm. The hemolysis rate was calculated using the following equation: hemolysis (%) = [(*A*_sample_ - *A*_PBS_)/(*A*_triton X-100_ - *A*_PBS_)] × 100. The experiment was performed in triplicate.

### 2.11 Cytotoxicity assay

Human skin keratinocyte HaCaT cells and mouse bone marrow-derived macrophages (BMDMs) were used to evaluate the cytotoxicity of Ply113. Cells were seeded in 96-well plates at 1 × 10^5^ cells per well and cultured for 12 h. Then the cells were exposed to different concentrations of Ply113 (ranging from 0 to 256 μg/mL) for 24 h. PBS buffer was used as a control. Cell viability was measured using the Cell Counting Kit-8 (CCK-8; Beyotime, Beijing, China), and absorbance was measured at 450 nm. The experiment was performed in triplicate.

### 2.12 Murine model of peritoneal septicemia

The procedure for the mouse experiment was approved by the Institutional Animal Care and Use Committee of the Harbin Veterinary Research Institute (Permission No. HVRI-IACUC-23110803GR). Peritoneal septicemia of mice was induced with *E. faecium*, *E. faecalis*, and *S. aureus* as previously described, with some modifications (Schuch et al., 2014; Brown Gandt et al., 2018). 6-week-old BALB/c mice were randomly assigned to five groups (*n* = 10 per group). The five groups were, respectively, inoculated intraperitoneally with 0.2 mL of *E. faecium* D73 (8 × 10^7^ CFU), *E. faecalis* E141 (7 × 10^7^ CFU), *S. aureus* YD2 (2 × 10^7^ CFU), *E. faecium*–*S. aureus* mixture (1:1 ratio), or *E. faecalis*–*S. aureus* mixture (1:1 ratio). After 1 h post-infection, five mice in each group were administered intraperitoneally with either 0.2 mL of Ply113 (10 mg/kg) or PBS. The mice were humanely euthanized after 24 h of infection, and the organs were homogenized with sterile PBS, and the bacterial loads in these organs were calculated by plate counting.

### 2.13 Statistical analysis

Statistical analysis was conducted and the graphs were created with GraphPad Prism 9.0 (GraphPad Software, Inc., La Jolla, CA, United States). All the data were assessed for normality using the Shapiro–Wilk test. The equality of variance was assessed using the F test or Brown-Forsythe test. If the data were normally distributed and had equal variance, an unpaired *t*-test or one-way analysis of variance (ANOVA) was used to determine statistical significance. A *p*-value of <0.05 was considered to be statistically significant.

## 3 Results

### 3.1 Lytic activity of Ply113 against *Enterococcus faecium* and *Enterococcus faecalis*

The bacteriophage endolysin Ply113 consists of 314 amino acids with an expected molecular weight of 34.1 kDa. According to SMART predictions, Ply113 has an N-terminal NlpC/P60 domain for enzymatic catalysis and a C-terminal SH3b domain for cell wall binding (Supplementary Figure S1A). The 3D structure of Ply113 was obtained by homology modeling using the crystal structure of the *Streptococcus pyogenes* bacteriophage endolysin PlyPy (PDB code: 5UDM) as a template. Molecular models and 3D structure analysis revealed that Ply113 has three α-helices and five β-sheets in the NlpC/P60 domain and seven β-sheets in the SH3 domain (Supplementary Figure S1B).

**Figure 1:**
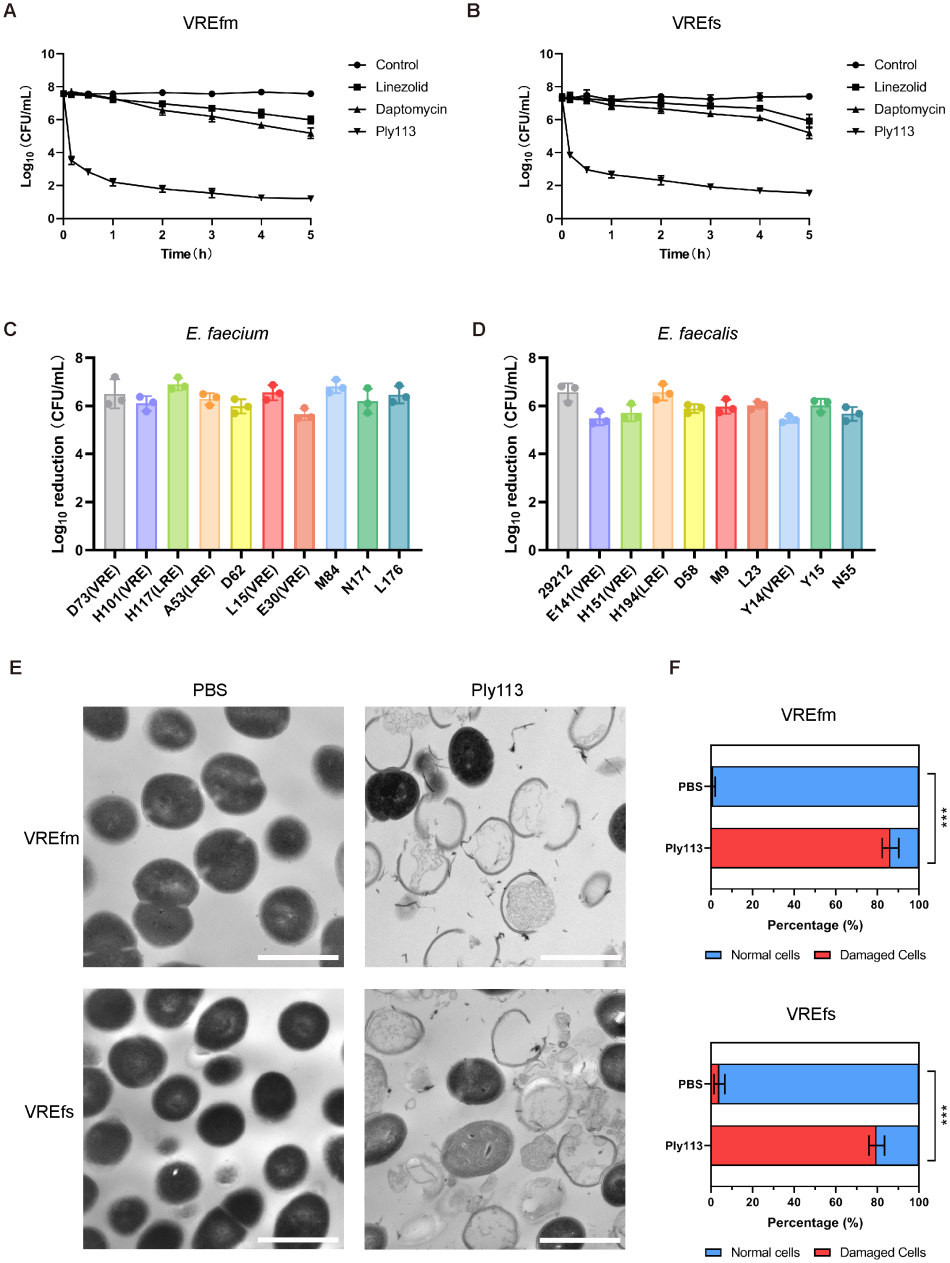
Lytic activity of Ply113 against *E. faecium* and *E. faecalis*. **(A,B)** Time-dependent killing curves of Ply113 and antibiotics against **(A)** VREfm strain ATCC700221 and **(B)** VREfs strain ATCC51299. Bacterial cells were incubated with Ply113 or antibiotics for different periods of time. The drug concentrations correspond to strain-specific 1 × MIC values. Each sample was diluted and spread onto BHI agar plates to count CFU. **(C,D)** Susceptibility of multiple isolates of **(C)** *E. faecium* and **(D)** *E. faecalis* to Ply113. An inoculum of approximately 10^8^ CFU/mL of different isolates was incubated with 8 μg/mL Ply113 at 37°C for 1 h. The reduction in bacterial CFU after Ply113 treatment indicates the bactericidal activity. **(E)** Morphological changes of VREfm and VREfs after exposure to Ply113. Scale bar, 1 μm. Cultures of VREfm and VREfs in the logarithmic growth phase were treated with 4 μg/mL Ply113 at 37°C for 1 h and observed using TEM. **(F)** The percentages of morphologically damaged cells of VREfm and VREfs after exposure to Ply113. Averages and standard deviations (SD) of three independent counts are shown. The number of cells for each count is 100 (*n* = 100). Statistical significance was calculated using unpaired *t* tests, ***p < 0.001.

The *in vitro* antibacterial activities of Ply113 against *E. faecium* and *E. faecalis* strains were examined. The MICs of Ply113 against these strains ranged from 4 to 8 μg/mL, as shown in Table 1. The lytic activity of Ply113 and antibiotics at their 1 × MIC were tested against *E. faecium* and *E. faecalis* strains. When vancomycin-resistant *E. faecium* (VREfm) strain ATCC700221 was treated with 4 μg/mL (1 × MIC) Ply113 for 10 min, log_10_ (CFU/mL) decreased by approximately 4.1; after 5 h, log_10_ (CFU/mL) decreased by nearly 6.4 (Figure 1A). Similarly, when vancomycin-resistant *E. faecalis* (VREfs) strain ATCC51299 was treated with 8 μg/mL (1 × MIC) Ply113 for 10 min, log_10_ (CFU/mL) decreased by 3.5, and after 5 h, log_10_ (CFU/mL) decreased by 5.8 (Figure 1B). Compared to Ply113, linezolid and daptomycin exhibited slower bactericidal activity. When VREfm and VREfs were treated with these antibiotics for 5 h, log_10_ (CFU/mL) reduced by no more than 2.5 (Figures 1A,B). The bacteriolytic spectrum of Ply113 against different *Enterococcus* isolates was also determined. As shown in Figures 1C,D, Ply113 was effective against all *E. faecium* and *E. faecalis* isolates tested, including VRE and linezolid-resistant *Enterococcus* (LRE).

**Table 1:** MICs of Ply113 and antibiotics against *Enterococcus faecium*, *Enterococcus faecalis*, and *Staphylococcus aureus*.

Strain^a^	MIC (μg/mL)				
	Ply113	Vancomycin	Daptomycin	Linezolid	Oxacillin
ATCC 700221 (VREfm)	4	128	2	2	–
D73 (VREfm)	4	128	1	1	–
ATCC 51299 (VREfs)	8	32	2	1	–
E141 (VREfs)	8	64	2	2	–
ATCC 29213 (MSSA)	8	1	1	1	1
ATCC 33591 (MRSA)	16	1	2	1	32
YD2 (MRSA)	16	2	1	1	32

a ATCC, American Type Culture Collection; VREfm, vancomycin-resistant *E. faecium*; VREfs, vancomycin-resistant *E. faecalis*; MRSA, methicillin-resistant *S. aureus*; MSSA, methicillin-susceptible *S. aureus*.

The morphological changes of VREfm and VREfs after treatment with Ply113 were observed using transmission electron microscopy (TEM). As shown in Figure 1E, VREfm and VREfs in PBS displayed a regular spherical shape, with intact cell walls and a homogeneous electron density in the cytoplasm. However, Ply113 treatment led to marked morphological damage in VREfm and VREfs. A majority of treated cells exhibited a fractured peptidoglycan structure and cytoplasmic membrane damage, along with the leakage of intracellular content from the rupture site. After Ply113 treatment, approximately 86.3% of VREfm cells were destroyed, whereas approximately 79.7% of VREfs cells were abnormal (Figure 1F). The percentage of damaged cells in treatment groups and untreated controls showed statistically significant differences (*p* < 0.001).

### 3.2 Lytic activity of Ply113 against *Staphylococcus aureus*

**Figure 2:**
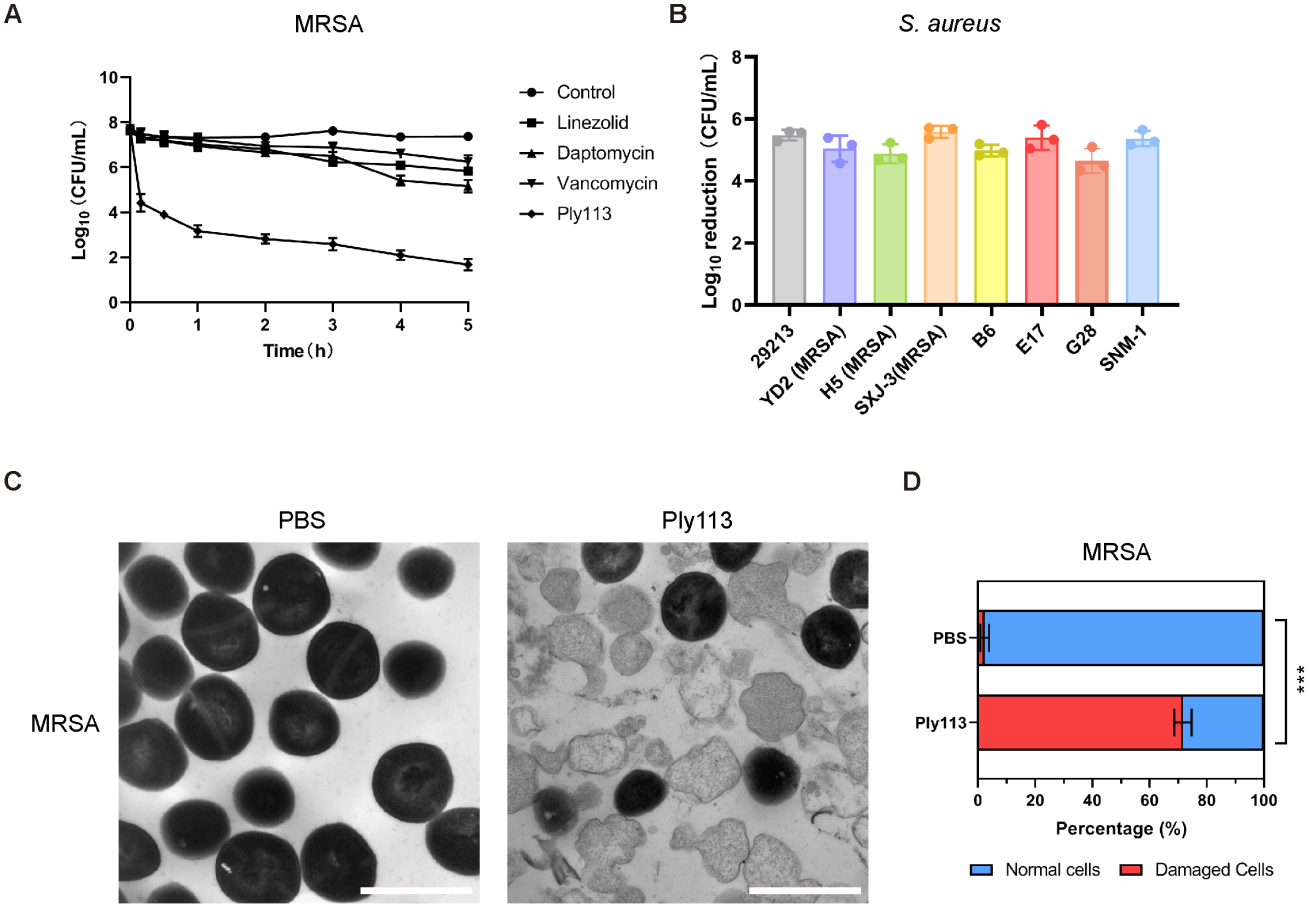
Lytic activity of Ply113 against *S. aureus*. **(A)** Time-dependent killing curves of Ply113 and antibiotics against MRSA strain ATCC33591. Bacterial cells were incubated with Ply113 or antibiotics for different periods of time. The drug concentrations correspond to strain-specific 1 × MIC values. Each sample was diluted and spread onto TSB agar plates to count CFU. **(B)** Susceptibility of multiple isolates of *S. aureus* to Ply113. An inoculum of approximately 10^8^ CFU/mL of different isolates was incubated with 8 μg/mL Ply113 at 37°C for 1 h. The reduction in bacterial CFU after Ply113 treatment indicates the bactericidal activity. **(C)** Morphological changes of MRSA after exposure to Ply113. Scale bar, 1 μm. Cultures of MRSA in the logarithmic growth phase were treated with 4 μg/mL Ply113 at 37°C for 1 h and observed using TEM. **(D)** The percentages of morphologically damaged cells of MRSA after exposure to Ply113. Averages ± SD of three independent counts are shown. The number of cells for each count is 100 (*n* = 100). Statistical significance was calculated using unpaired *t* tests, ***p < 0.001.

The MICs of Ply113 against *S. aureus* strains ranged from 8 to 16 μg/mL, as shown in Table 1. The lytic activities of Ply113 against *S. aureus* strains were further tested in a time-killing assay. As shown in Figure 2A, Ply113 was highly active against MRSA ATCC33591. After treatment with 16 μg/mL (1 × MIC) Ply113 for 10 min, log_10_ (CFU/mL) decreased by 3.3. After treatment for 5 h, log_10_ (CFU/mL) decreased by 6.0 (Figure 2A). Log_10_ (CFU/mL) dropped by no more than 2.6 when MRSA was treated with linezolid, daptomycin, and vancomycin for 5 h. Ply113 was also shown to be active against a variety of *S. aureus* isolates, including MRSA isolates, with log_10_ (CFU/mL) decreasing by 4.7–5.6 (Figure 2B). TEM observation revealed that MRSA cells exposed to Ply113 exhibited substantial morphological alterations (Figure 2C). Untreated MRSA cells had a smooth outer layer of normal cells with a typical ball shape. Ply113 treatment caused cell wall detachment in MRSA, exhibiting a deep distortion and rupture of the cytoplasmic membrane. The electron density in the cytoplasm was drastically reduced, and many cells were lysed like “ghost cells.” Nearly 71.7% of cells were damaged after Ply113 treatment (Figure 2D). The percentage of damaged cells in the treatment and control groups differed statistically significantly (*p* < 0.001).

### 3.3 Biochemical characterization and stability of Ply113

**Figure 3:**
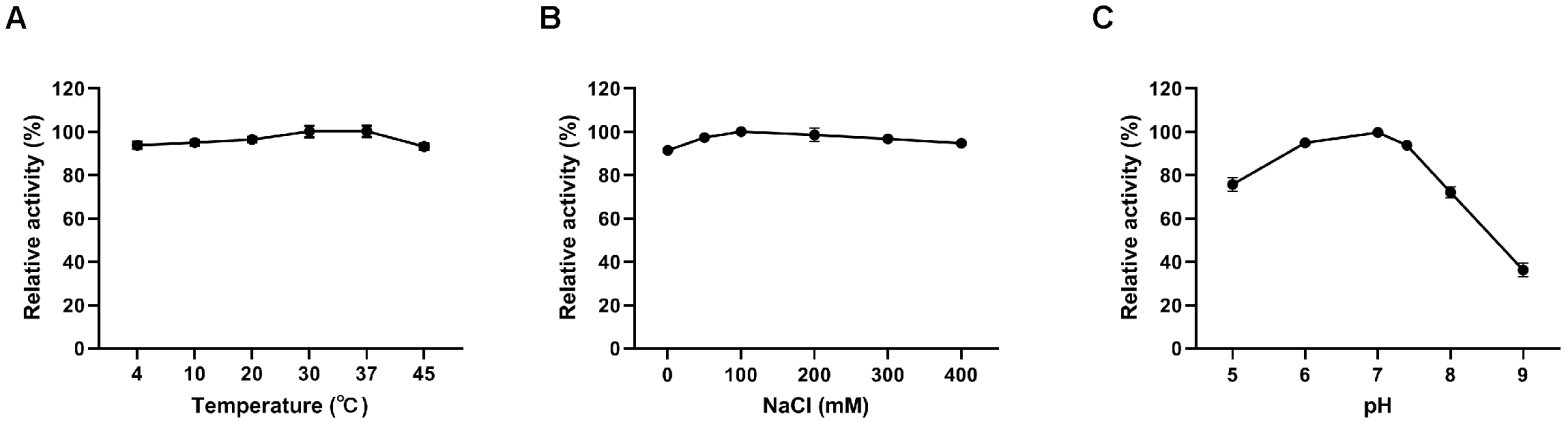
Effects of different conditions on the lytic activity of Ply113. The effects of **(A)** temperature, **(B)** NaCl, and **(C)** pH on the lytic activity of Ply113 were examined by incubating bacterial suspensions of *E. faecalis* with 4 μg/mL Ply113 under different conditions for 1 h. The maximum activity of Ply113 under each test condition was set as 100%. Data are presented as the mean ± SD from three independent tests.

The influence of temperature, salinity, and pH on the lytic activity of Ply113 was investigated by assessing CFU. Ply113 remained relatively stable, with high activity at temperatures ranging from 4°C to 45°C (Figure 3A). The effect of salt concentration on the lytic activity of Ply113 was also minimal. Ply113 exhibited the highest lytic activity when the salt concentration was 100 mM. As the concentration was increased to 400 mM, the lytic activity of Ply113 decreased marginally (Figure 3B). Although the lytic activity of Ply113 declined to 36.2% at pH 9.0, it maintained a high lytic activity over a wide pH range of 5.0–8.0 (Figure 3C).

### 3.4 Antibiofilm activity of Ply113 against the mono-species biofilms of *Enterococcus faecium*, *Enterococcus faecalis*, and *Staphylococcus aureus*

**Figure 4:**
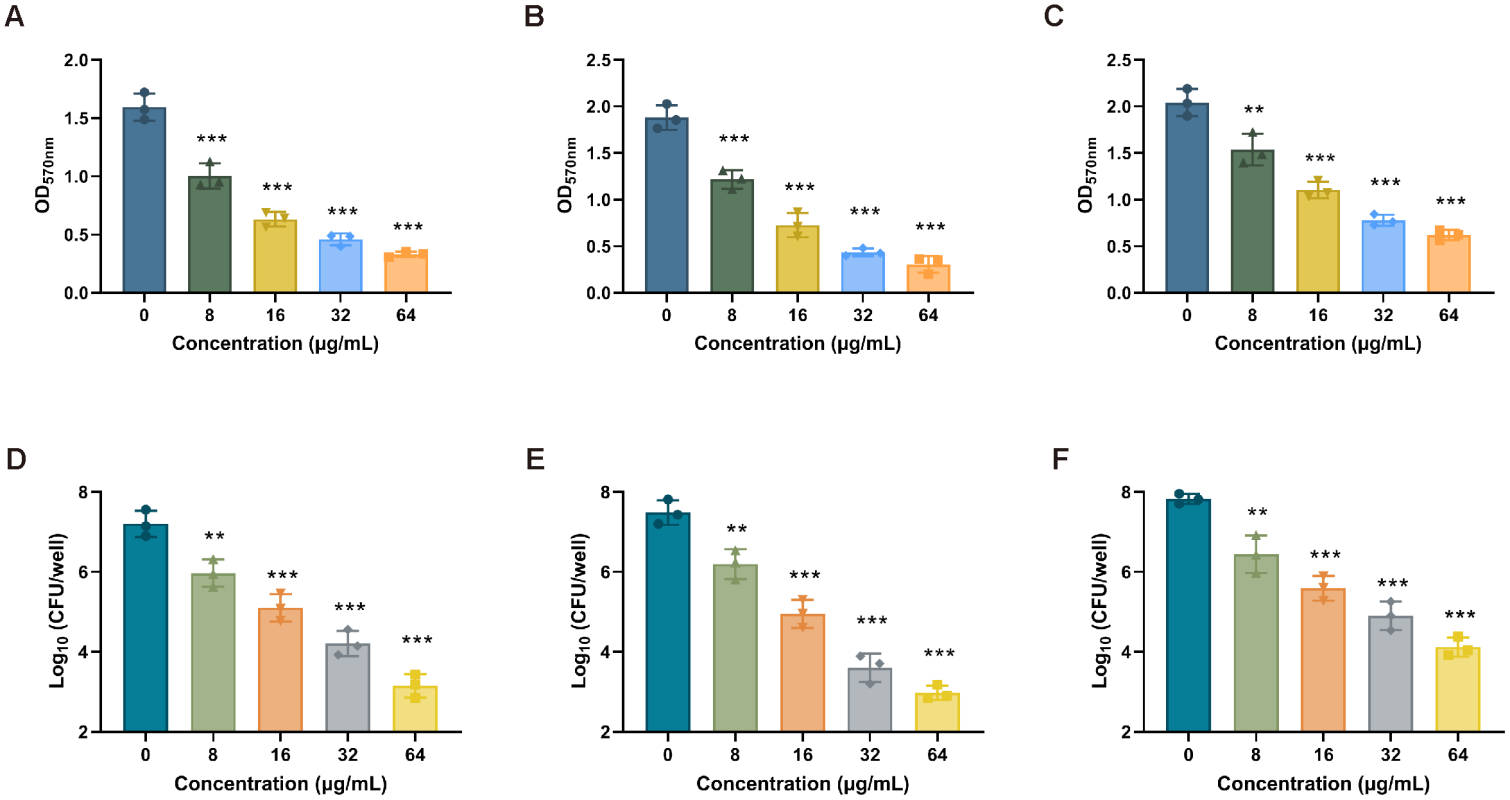
Biofilm removal efficacy of Ply113 against the mono-species biofilms. **(A–C)** Effectiveness of Ply113 on the mono-species biofilms of **(A)** *E. faecium*, **(B)** *E. faecalis*, and **(C)** *S. aureus* preformed in 24-well plates. The preformed mono-species biofilms were treated with different concentrations of Ply113 at 37°C for 2 h and quantified using crystal violet staining. **(D–F)** Quantifications of **(D)** *E. faecium*, **(E)** *E. faecalis*, and **(F)** *S. aureus* by CFU counting after Ply113 treatment of preformed mono-species biofilms. Data are presented as the mean ± SD from three independent tests. Statistical significance was calculated using one-way ANOVA. **p < 0.01, ***p < 0.001.

To determine the antibiofilm activity of Ply113, the mono-species biofilms of *E. faecium*, *E. faecalis*, and *S. aureus* were formed in 24-well polystyrene microplates, and each well was exposed to Ply113 for 2 h. The VREfm isolate D73, VREfs isolate E141, and MRSA isolate YD2 were chosen for the biofilm elimination assay on the basis of their capacity to form strong biofilms. The crystal violet staining results showed that Ply113 could significantly eliminate the biofilms of *E. faecium*, *E. faecalis*, and *S. aureus* in a dose-dependent manner (Figures 4A–C). Following treatment with 64 μg/mL Ply113, approximately 91.7, 95.8, and 78.7% of the biofilms formed by *E. faecium*, *E. faecalis*, and *S. aureus*, respectively, were eliminated. The number of culturable bacteria in biofilms after exposure to Ply113 was also evaluated. As shown in Figures 4D–F, the 24-h biofilms contained 10^7.2^ CFU/mL of *E. faecium*, 10^7.5^ CFU/mL of *E. faecalis*, and 10^7.8^ CFU/mL of *S. aureus*. After 2 h of Ply113 treatment, the numbers of culturable bacteria in mono-species biofilms decreased with increasing Ply113 concentrations. *E. faecalis* was the most sensitive to Ply113; log_10_ (CFU/mL) was reduced by 4.5 when 64 μg/mL of Ply113 was applied. Under the same concentration of Ply113, log_10_ (CFU/mL) was reduced by 4.1 in *E. faecium* biofilms and by 3.7 in *S. aureus* biofilms. These results suggest that Ply113 can disrupt the mono-species biofilms of *E. faecium*, *E. faecalis*, and *S. aureus*.

### 3.5 Capability of Ply113 to eliminate dual-species biofilms produced by *Enterococcus* species and *Staphylococcus aureus*

**Figure 5:**
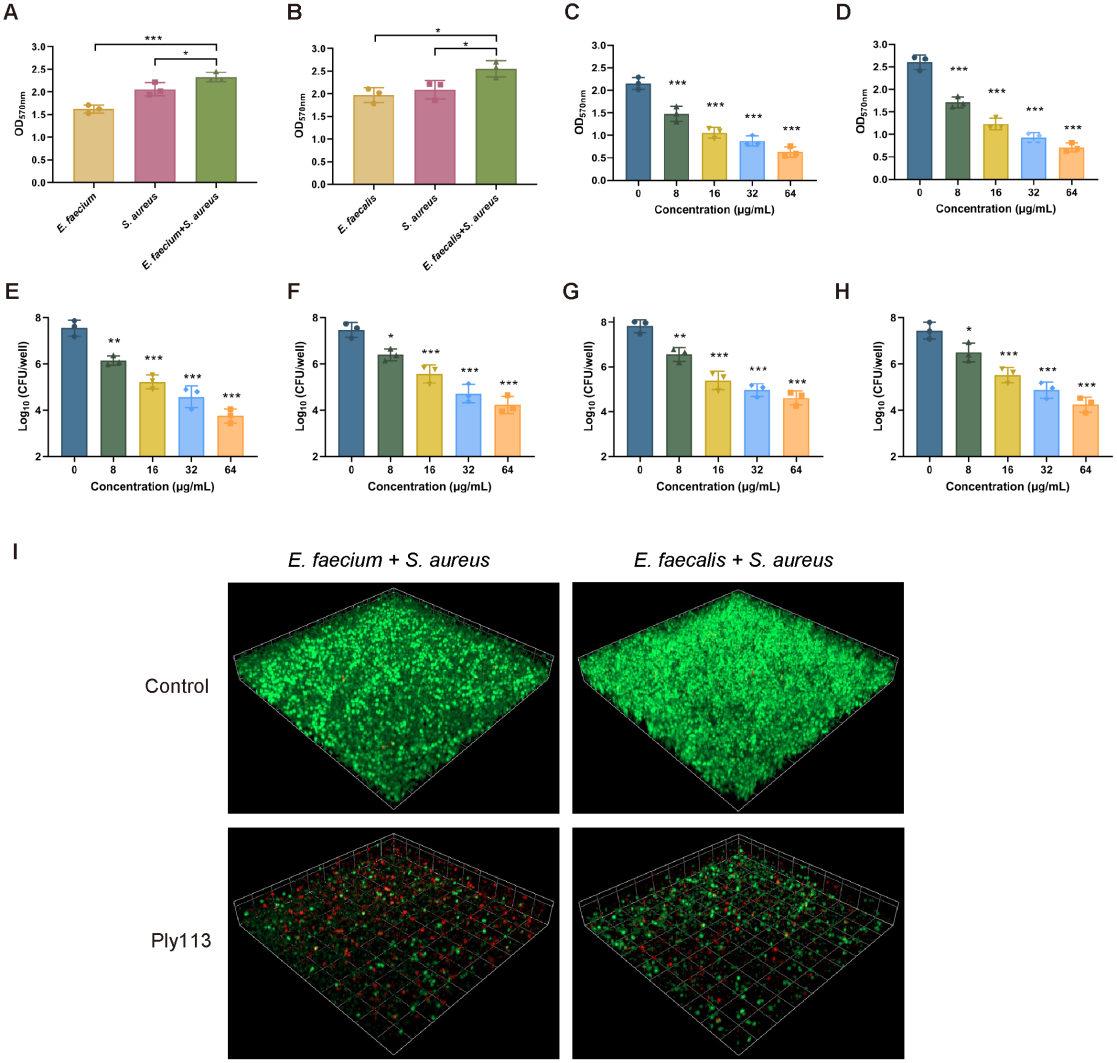
Biofilm elimination efficacy of Ply113 against dual-species biofilms. **(A,B)** Dual-species biofilms formed in 24 h by either **(A)** *E. faecium*–*S. aureus* or **(B)** *E. faecalis*–*S. aureus* at a 1:1 ratio in 24-well plates. **(C,D)** Effects of Ply113 on the dual-species biofilms of **(C)** *E. faecium*–*S. aureus* and **(D)** *E. faecalis*–*S. aureus*. The preformed dual-species biofilms were incubated with various concentrations of Ply113 at 37°C for 2 h and quantified using crystal violet staining. **(E–H)** Quantifications of **(E)** *E. faecium* and **(F)** *S. aureus* in the *E. faecium*–*S. aureus* biofilms and **(G)** *E. faecalis* and **(H)** *S. aureus* in the *E. faecalis*–*S. aureus* biofilms by CFU counting after Ply113 treatment. **(I)** Confocal micrographs of SYTO 9–PI-stained dual-species biofilms of *E. faecium*–*S. aureus* and *E. faecalis*–*S. aureus* after Ply113 treatment for 2 h. Data are presented as the mean ± SD from three independent tests. Statistical significance was calculated using one-way ANOVA. *p < 0.05, **p < 0.01, ***p < 0.001.

To produce dual-species biofilms, *E. faecium* or *E. faecalis* was mixed with *S. aureus* in polystyrene microplates. When *E. faecium* and *S. aureus* were cultured together for 24 h, greater biofilm production was observed compared with each mono-species biofilm (Figure 5A). The biofilm production between the dual-species biofilms and mono-species biofilms differed statistically significantly (*p* < 0.05). A similar observation was made in the dual-species biofilms produced by *E. faecalis* and *S. aureus* (Figure 5B). Preformed dual-species biofilms were treated with Ply113 for 2 h and analyzed using crystal violet staining. Ply113 eliminated the dual-species biofilms of *E. faecium*–*S. aureus* and *E. faecalis*–*S. aureus* in a dose-dependent manner (Figures 5C,D). Ply113 at a concentration of 64 μg/mL eliminated approximately 78.3% of the *E. faecium*–*S. aureus* biofilms and 79% of the *E. faecalis*–*S. aureus* biofilms. The number of culturable bacteria in dual-species biofilms reduced as Ply113 concentrations increased (Figures 5E–H). To visualize the efficacy of Ply113 in disrupting the dual-species biofilms, SYTO 9–PI staining was performed on 24-h-old dual-species biofilms following exposure to 8 μg/mL Ply113. Ply113 treatment of *E. faecium*–*S. aureus* or *E. faecalis*–*S. aureus* dual-species biofilms resulted in a considerable decrease in SYTO 9-stained green bacterial cells and a significant increase in PI-stained red dead cells (Figure 5I). These results suggest that Ply113 has a high biofilm-disrupting potential not only against mono-species but also against polymicrobial biofilms.

### 3.6 Hemolytic activity and cytotoxicity of Ply113

**Figure 6:**
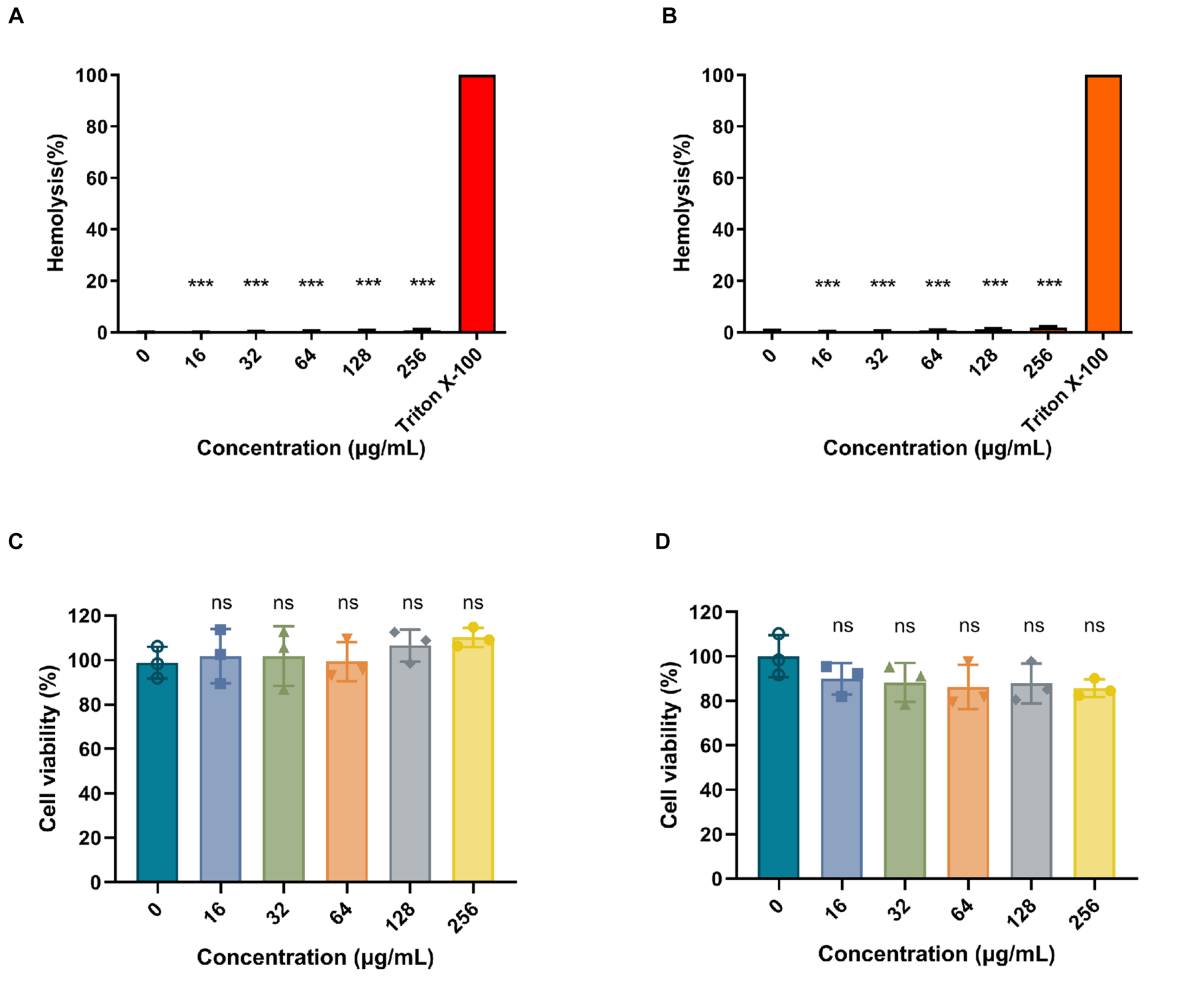
Hemolytic activity and cytotoxicity of Ply113. **(A,B)** Hemolytic activities of Ply113 on **(A)** pig red blood cells (RBCs) and **(B)** rabbit RBCs. RBCs were incubated with different concentrations of Ply113 at 37°C for 1 h. Hemolysis was determined by measuring the absorbance at 545 nm. PBS served as a negative control, Triton X-100 served as a positive control. **(C,D)** Cytotoxicity of Ply113 on **(C)** HaCaT cells and **(D)** BMDMs. Cell viability was examined after incubating cell suspensions with different concentrations of Ply113 for 24 h. Data are presented as the mean ± SD from three independent tests. Statistical significance was calculated using one-way ANOVA. ***p < 0.001, ns, not significant.

To determine the hemolytic activity of Ply113, hemolytic assays were conducted by incubating RBCs from pigs and rabbits with Ply113. As shown in Figures 6A,B, Ply113 was found to have negligible hemolytic activity (<2%) in either pig or rabbit RBCs, even at concentrations of up to 256 μg/mL. Hemolysis in the treatment and positive control groups differed statistically significantly (*p* < 0.001). The potential cytotoxicity of Ply113 was also evaluated by performing the CCK-8 assay using HaCaT cells and BMDMs. No significant reduction (*p* > 0.05) in cell viability was detected after 24 h of Ply113 treatment at concentrations ranging from 16 to 256 μg/mL, but a 10–14.3% drop in the cell viability of mouse BMDMs was observed (Figures 6C,D).

### 3.7 *In vivo* antibacterial efficacy of Ply113

**Figure 7:**
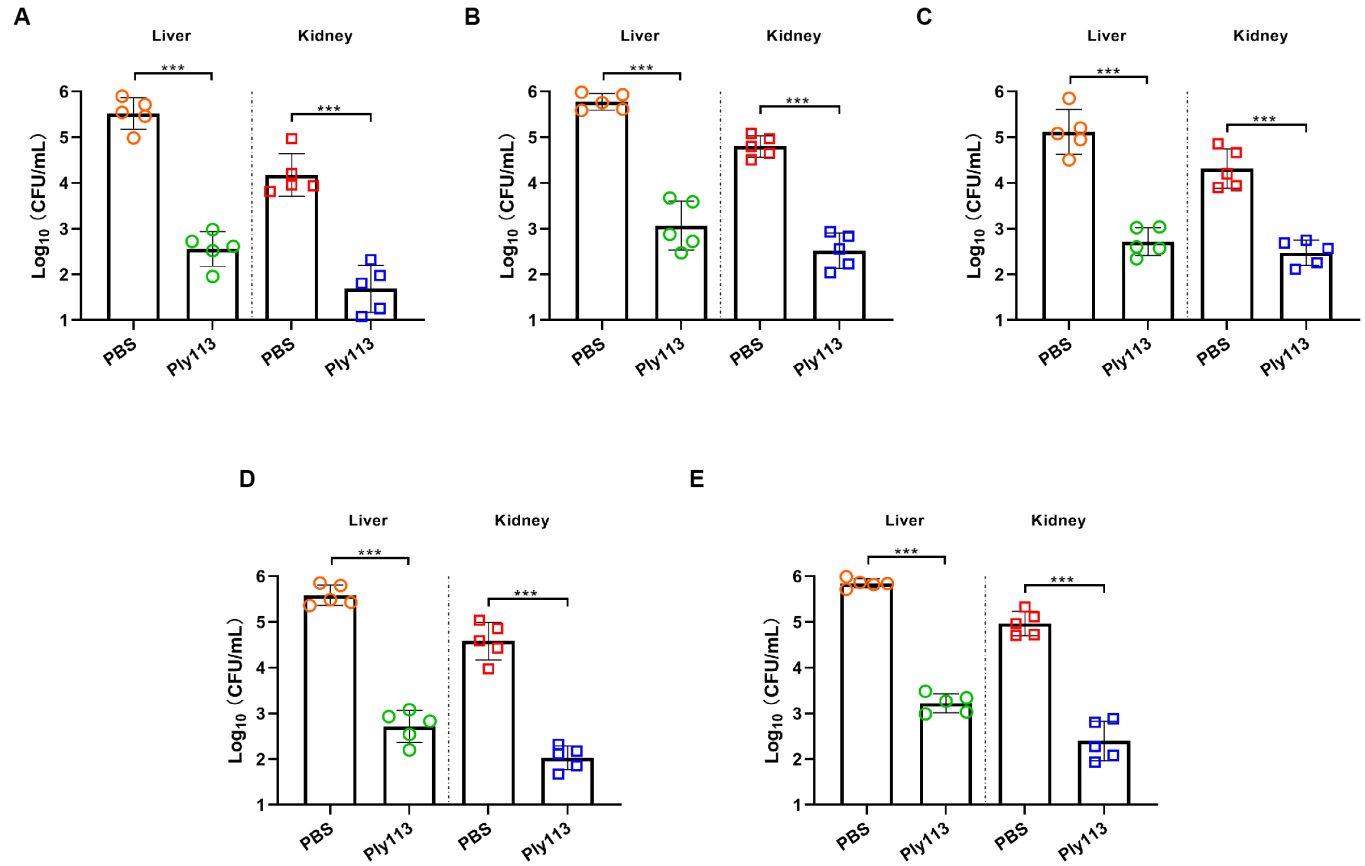
*In vivo* antibacterial efficacy of Ply113 in a murine model of peritoneal septicemia. BALB/c mice were inoculated intraperitoneally with **(A)** *E. faecium*, **(B)** *E. faecalis*, **(C)** *S. aureus*, **(D)** the *E. faecium*–*S. aureus* mixture, or **(E)** the *E. faecalis*–*S. aureus* mixture, and then treated with Ply113 1 h later. After 24 h of treatment, samples were collected from liver and kidney for bacterial quantification. Values represent the mean ± SD from five mice. Statistical significance was calculated using unpaired *t* tests. ***p < 0.001.

The *in vivo* efficacy of Ply113 was evaluated in a murine model of peritoneal septicemia. Mice were, respectively, infected with *E. faecium*, *E. faecalis*, *S. aureus*, the *E. faecium–S. aureus mixture*, or the *E. faecalis–S. aureus mixture*, and then treated with either Ply113 or PBS 1 h later. As shown in Figures 7A–C, the bacterial loads in the livers and kidneys of Ply113-treated mice decreased by 3.0 and 2.5 log units in the *E. faecium*-infected group, 2.7 and 2.3 log units in the *E. faecalis*-infected group, and 2.4 and 1.8 log units in the *S. aureus*-infected group, respectively. In addition, Ply113 treatment was also found to reduce the bacterial loads (>2.5 log units) in the livers and kidneys of the *E. faecium*–*S. aureus* and *E. faecalis*–*S. aureus* infected groups using this single dose (Figures 7D,E). The bacterial loads in the organs between the treatment and control groups differed statistically significantly (*p* < 0.001).

## 4 Discussion

The Gram-positive pathogenic bacteria *E. faecium*, *E. faecalis*, and *S. aureus* are internationally recognized as the three most concerning antibiotic-resistant pathogens causing hospital and community-related infections (Huang et al., 2019; Weiner-Lastinger et al., 2020). These three pathogenic bacteria can infect both humans and animals and cause sepsis, mastitis, and other diseases, threatening public health globally (Fitzgerald, 2012; Abat et al., 2016; Argudin et al., 2017). In recent years, the evolution of antibiotic resistance has become increasingly serious. The prevalence of MRSA and VRE is still steadily increasing, while vancomycin-resistant *S. aureus* and daptomycin-resistant *Enterococcus* are emerging (Munoz-Price et al., 2005; Cong et al., 2020). The antibiotic resistance crisis has highlighted the urgent need for innovation in therapeutics and treatment strategies.

Bacteriophage-derived endolysins are considered to have significant potential as novel therapeutic agents for effectively combating antibiotic-resistant bacterial infections. In the present study, the endolysin Ply113 from an *E. faecium* bacteriophage was identified and expressed, and its antibacterial activity was evaluated. Ply113 endolysin was chosen after searching for endolysin domains in the National Center for Biotechnology Information database (Oliveira et al., 2013), and we discovered that it had outstanding antibacterial activity against some Gram-positive bacteria. Ply113 showed high lytic activity against a broad range of *E. faecium* and *E. faecalis* strains, including clinical isolates of VRE and LRE. Ply113 also exhibited cross-species bactericidal efficacy against *S. aureus* strains, including MRSA isolates. Bacteriophages typically have species specificity, but several phage endolysins exhibit cross-species lytic activity. For example, the endolysin PlySs2 was found to have lytic activity against MRSA as well as a variety of *Streptococcus* species (Gilmer et al., 2013). Similarly, the LysSS endolysin has been shown to have activity against *Acinetobacter baumannii*, *Klebsiella pneumoniae*, *Pseudomonas aeruginosa*, and other bacterial species (Kim et al., 2020). Similar to PlySs2 and LysSS, Ply113 has cross-species lytic activity and is highly effective against both MRSA and VRE, which are notorious antibiotic-resistant pathogens. This relatively wide lytic spectrum of Ply113 also enhanced the possibility of its therapeutic application for the treatment of infections caused by Gram-positive pathogens.

An issue that cannot be ignored is that biofilms play an important role in bacterial infections, accounting for nearly 80% of human microbial infections (Romling and Balsalobre, 2012). Thus, the choice of antibacterial therapies also needs to consider the effectiveness of biofilm eradication. Endolysins have been recommended as a promising antimicrobial alternative for controlling bacterial biofilms (Liu et al., 2023). LysSA52, an endolysin derived from an *S. aureus* bacteriophage, was reported to remove 60% of biofilms after 12 h of treatment at a concentration of 500 μg/mL (Abdurahman et al., 2023). Another study showed that the Lys84 endolysin at concentrations greater than 10 μM could effectively eliminate 90% of the biofilms of *S. aureus* in 2 h (Ning et al., 2021). According to the present findings, Ply113 at concentrations of more than 8 μg/mL (0.24 μM) was capable of significantly eliminating biofilms of *E. faecium*, *E. faecalis*, and *S. aureus* after 2 h of treatment. At a Ply113 concentration of 64 μg/mL (1.9 μM), up to 95.8% of the biofilm could be eliminated, demonstrating the high efficiency of Ply113 in biofilm removal.

**Figure 8:**
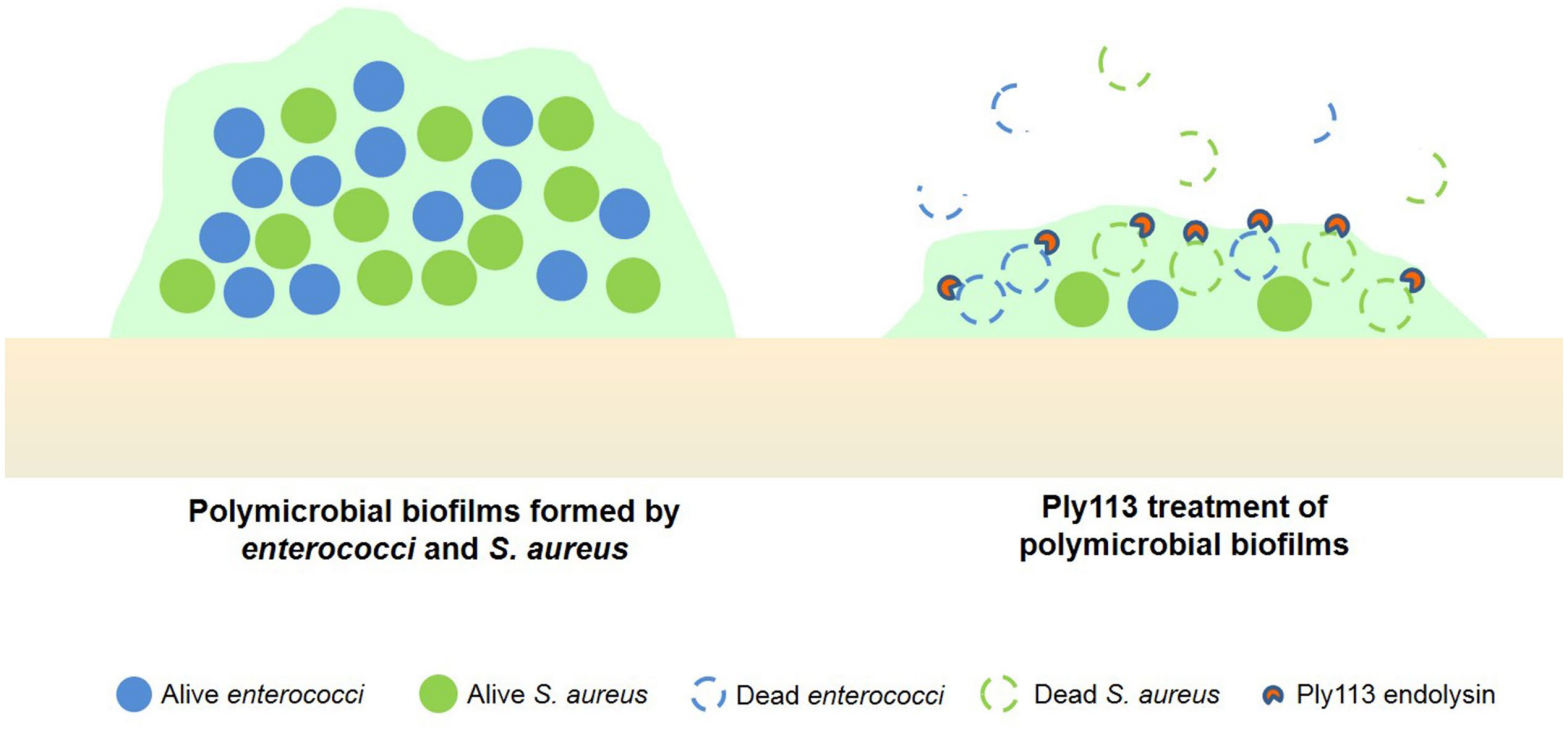
Ply113 treatment of dual-species biofilms formed by *enterococci* and *S. aureus*. Ply113 exerts rapid and high lytic activity against strains of *E. faecium*, *E. faecalis*, and *S. aureus*, and eliminates the dual-species biofilms of *E. faecium*–*S. aureus* and *E. faecalis*–*S. aureus.*

In clinical settings, it is common for multiple bacterial species to co-infect and invade the same tissue in a host (Pammi et al., 2014; Azevedo et al., 2017). The formation of polymicrobial biofilms involves two or more bacterial species interacting with each other, enhancing their antibiotic resistance and pathogenicity through a variety of mechanisms (Sadiq et al., 2022). Here, we observed a significant increase in biofilm formation when *E. faecalis* was co-cultured with *S. aureus*, in comparison to individual cultures. A previous study has shown that interactions between *E. faecalis* and *S. aureus* could promote the formation of dual-species biofilms (Ch'ng et al., 2022), which is consistent with our findings. In addition, we observed that dual-species biofilm formation was also enhanced in the combination of *E. faecium*–*S. aureus*. Several phage endolysins have been demonstrated to be efficient against bacterial biofilms (Poonacha et al., 2017; Ning et al., 2021; Abdurahman et al., 2023; Liu et al., 2023); however, these antibiofilm effects have largely been verified on mono-species biofilms. Polymicrobial biofilms are more tolerant to antimicrobial agents than mono-species biofilms; for example, ciprofloxacin concentrations must be higher against polymicrobial biofilms formed by *S. aureus* and *P. aeruginosa* compared with mono-species biofilms (Tkhilaishvili et al., 2020). Therefore, the original therapy dose may be ineffective in eliminating polymicrobial biofilms, which is also the main reason why polymicrobial infections often result in treatment failure and even death (Pammi et al., 2014). Several antibacterial agents have recently been reported for their action on polymicrobial biofilms formed by *enterococci* and *S. aureus*. It has been discovered that the bacteriophage phiIPLA-RODI can kill *S. aureus* but not *E. faecium* in polymicrobial biofilms (Gonzalez et al., 2017). Additionally, the cationic peptide Brevinin2 HYba5 was found to inhibit the biofilm formation of *S. aureus* and *E. faecalis* individually as well as in polymicrobial biofilm; however, its concentration is up to 75 μM (Radhakrishnan et al., 2023), which may be too high to reduce its therapeutic value. In the present study, we found that Ply113 at a concentration of 64 μg/mL (1.9 μM) was able to remove the majority of the mono-species biofilms of *E. faecium*, *E. faecalis*, and *S. aureus*. When Ply113 was applied to dual-species biofilms at the same concentration, it eliminated the biofilms with equivalent efficiency. Our findings imply that Ply113 has the potential to be a valuable antimicrobial agent against polymicrobial infections (Figure 8).

In summary, our findings show that bacteriophage endolysin Ply113 can be applied to effectively decrease the bacterial burden of *E. faecium*, *E. faecalis*, and *S. aureus in vitro* and *in vivo*. Furthermore, Ply113 exerts the potent bactericidal activity against polymicrobial biofilms formed by *enterococci* and *S. aureus*. This makes Ply113 is a promising therapeutic candidate for the treatment of polymicrobial infections and biofilm-associated infections, such as chronic and recurrent infections. We are currently exploring the combination of Ply113 and other drugs, including antibiotics and antimicrobial peptides, to improve their therapeutic efficacy. Although not statistically significant, there was a more than 10% decrease in the cell viability of mouse BMDMs, and the *in vivo* safety of Ply113 still needs to be further validated. Whether Ply113 therapy can be used in the future also still needs to be further validated using *in vivo* models, such as skin wound infections and catheter-related biofilm infections.

## Data availability statement

The datasets presented in this study can be found in online repositories. The names of the repository/repositories and accession number(s) can be found in the article/Supplementary material.

## Ethics statement

The animal study was approved by the Animal Ethics Committee of the Harbin Veterinary Research Institute of the Chinese Academy of Agricultural Sciences. The study was conducted in accordance with the local legislation and institutional requirements.

## Author contributions

JW: Conceptualization, Methodology, Writing – original draft. SLia: Methodology, Validation, Writing – original draft. XL: Data curation, Writing – original draft. QX: Investigation, Writing – original draft. YZ: Resources, Writing – original draft. SY: Software, Writing – original draft. WZ: Supervision, Writing – review & editing. SLiu: Project administration, Writing – review & editing. FX: Conceptualization, Funding acquisition, Writing – review & editing.
