# Supplementary material for: Bacteriophage endolysin Ply113 as a potent antibacterial agent against polymicrobial biofilms formed by enterococci and Staphylococcus aureus
Source: Front Microbiol. 2023 Dec 12;14:1304932. doi: 10.3389/fmicb.2023.1304932 (PMC10751913; doi:10.3389/fmicb.2023.1304932)
Supplement: Supplementary file 1 [file Table_1.DOCX]

**Table 1**

**Bacterial strains used in this study**

| **Species** | **Strains** | **Origin** |
| --- | --- | --- |
| *Enterococcus faecium* | 700221 | ATCC |
|  | D73 | Human |
|  | H101 | Human |
|  | H117 | Human |
|  | A53 | Human |
|  | D62 | Swine |
|  | L15 | Swine |
|  | E30 | Bovine |
|  | M84 | Bovine |
|  | N171 | Human |
|  | L176 | Human |
| *Enterococcus faecalis* | 29212 | ATCC |
|  | 51299 | ATCC |
|  | E141 | Human |
|  | H151 | Human |
|  | H194 | Human |
|  | D58 | Swine |
|  | M9 | Swine |
| *Enterococcus faecalis* | L23 | Bovine |
|  | Y14 | Bovine |
|  | Y15 | Bovine |
|  | N55 | Bovine |
| *Staphylococcus aureus* | 29213 | ATCC |
|  | YD2 | Human |
|  | H5 | Bovine |
|  | SXJ-3 | Human |
|  | B6 | Bovine |
|  | E17 | Swine |
|  | G28 | Swine |
|  | SNM-1 | Human |
