# Supplementary material for: Bacteriophage endolysin Ply113 as a potent antibacterial agent against polymicrobial biofilms formed by enterococci and Staphylococcus aureus
Source: Front Microbiol. 2023 Dec 12;14:1304932. doi: 10.3389/fmicb.2023.1304932 (PMC10751913; doi:10.3389/fmicb.2023.1304932)
Supplement: Supplementary file 2 [file Image_1.pdf]

A

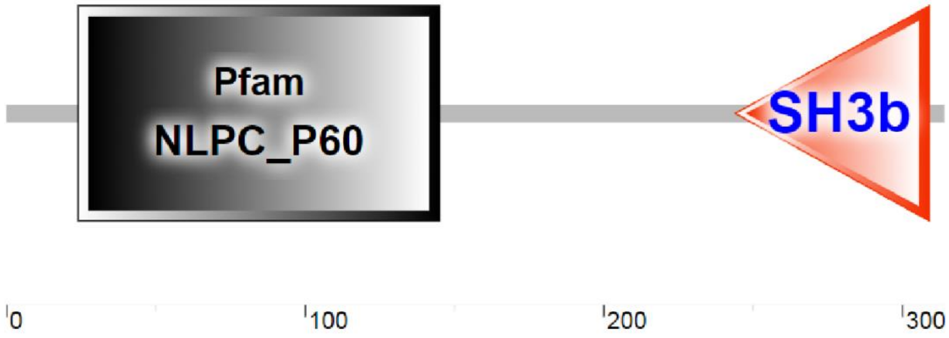

B

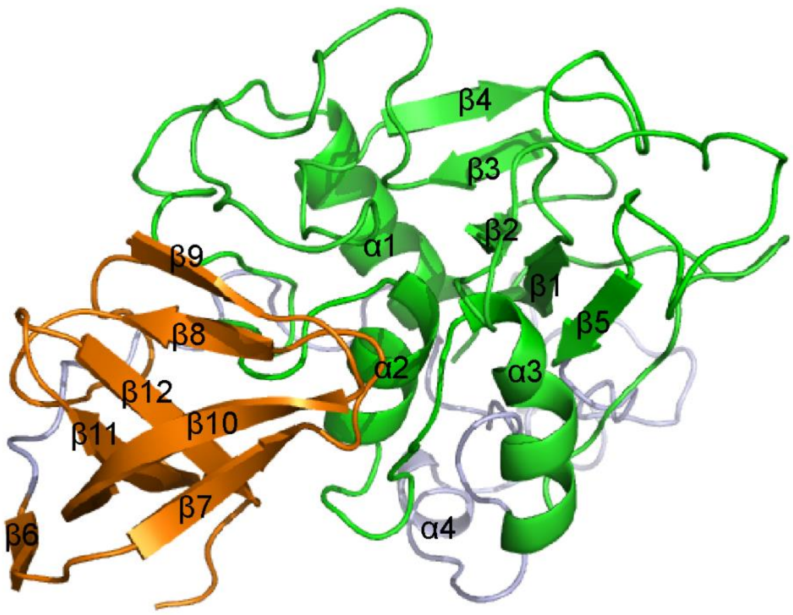

Supplementary Figure S1. General features of Ply113.

(A) Protein domains of Ply113 identified using SMART. (B) Three-dimensional structural model of Ply113. The N-terminal NlpC/P60 domain is shown in green, and the C-terminal SH3b domain is shown in orange. Protein structure homology modeling was conducted with SWISS-MODEL using the crystal structure of the *S. pyogenes* bacteriophage endolysin PlyPy (PDB code: 5UDM) as a template. Results are presented by PyMOL.
